# Correlation-Based Network Generation, Visualization, and Analysis as a Powerful Tool in Biological Studies: A Case Study in Cancer Cell Metabolism

**DOI:** 10.1155/2016/8313272

**Published:** 2016-10-19

**Authors:** Albert Batushansky, David Toubiana, Aaron Fait

**Affiliations:** ^1^The Jacob Blaustein Institutes for Desert Research, Ben-Gurion University of the Negev, 84990 Midreshet Ben-Gurion, Israel; ^2^Telekom Innovation Laboratories, Department of Information Systems Engineering, Ben-Gurion University of the Negev, 84105 Beer Sheva, Israel

## Abstract

In the last decade vast data sets are being generated in biological and medical studies. The challenge lies in their summary, complexity reduction, and interpretation. Correlation-based networks and graph-theory based properties of this type of networks can be successfully used during this process. However, the procedure has its pitfalls and requires specific knowledge that often lays beyond classical biology and includes many computational tools and software. Here we introduce one of a series of methods for correlation-based network generation and analysis using freely available software. The pipeline allows the user to control each step of the network generation and provides flexibility in selection of correlation methods and thresholds. The pipeline was implemented on published metabolomics data of a population of human breast carcinoma cell lines MDA-MB-231 under two conditions: normal and hypoxia. The analysis revealed significant differences between the metabolic networks in response to the tested conditions. The network under hypoxia had 1.7 times more significant correlations between metabolites, compared to normal conditions. Unique metabolic interactions were identified which could lead to the identification of improved markers or aid in elucidating the mechanism of regulation between distantly related metabolites induced by the cancer growth.

## 1. Introduction

Advanced technology methods for high-throughput biological studies, such as metabolomics and transcriptomics developed during the last decades, are successfully applied in biomedical research [1], plant studies [2], and microbiology [3]. The wide use of these technologies led to the accumulation of data on biological processes at their multiple levels (metabolic, genetic, enzymatic, physiological, phenotypical, etc.) and called for the development of tools to ease the visualization, analysis, and interpretation of an often complex and multidimensional matrix. Furthermore, the readily available “*omics*” technologies in biological laboratories prompted biologists to enter a field often needing extensive computational knowhow and led to the increased interest in biological interaction networks [4]. Thus, in the recent decades networks describing cellular processes were generated for human [5], yeast [6], and plants [7].

Networks can be presented as graphs, that is, a set of vertices (V) connected by edges (E), and consequently can be analyzed using graph theory, an approach that has been increasingly implemented in biological studies during the last decade. It is commonly accepted that graph theory as a scientific discipline was first used by the Swiss mathematician Leonhard Euler in 1735-1736, tackling the Königsberg bridge problem. Later, in the 19th and 20th centuries, graph theory was formulated and eventually introduced for applied fields, such as physics, computer science, and biology [8]. Today, graph theory consists of many tens of basic definitions and properties [9]. The understanding of the biological networks lies in the nature of the vertices and edges between them; that is, the vertices may represent one of the components of the three major molecular levels: genes, proteins, or metabolites, while the edges between them represent gene coexpression, protein-protein interactions, or biochemical conversions of metabolites, respectively [10]. However, molecular networks are not delimited to illustrate single-level component interactions. They can also show cross-level interactions. Alternatively, and perhaps a little counterintuitive, a network may incorporate vertices representing a set of metabolic reactions, where the connection between a pair of vertices is established if the reactions share one or multiple metabolites used or produced by these reactions [11, 12]. In other networks, vertices represent a community of molecular components, especially used with very vast data sets (>1000 of components) such as in weighted gene coexpression network analysis (WGCNA). Here, a single vertex delineates a module of genes and edges between vertices represent the correlation between them. This allows reducing the complexity of the network and simultaneously retains most of the information used for the interpretation of the gene coexpression results [13]. In simple words, vertices and edges represent the information as defined by the creator/user of the network.

In the last decade, correlation-based network analysis (CNA) has become a popular data-mining tool for visualizing and analyzing biological relationships within large data sets [13, 14]. In this type of networks, vertices and edges represent molecular elements (e.g., metabolites or genes) and their correlation coefficient (strength and sign), respectively [10, 15, 16]. Edges inferred by correlation analyses reflect a coordinated behavior between vertices across the data set (treatments, genotypes, conditions, and time). The type of correlation has to be selected based on the parametrical distribution of the data. In large population studies, data has to be tested for normality using existing tests, for example, the Shapiro-Wilk test. The Pearson correlation should be applied to normally distributed data, while Spearman's rank correlation should be used for data violating the assumption of normal distribution. CNA was successfully applied to various biological systems; it revealed, for example, metabolic markers related to plant growth and biomass in* Arabidopsis thaliana* recombinant inbred lines (RIL) and introgression lines (IL) [17, 18], the role of gene Col5a2 in myocardial infarction [19], effect of hypoxia on tumor cell biochemistry [20], and recently, identification of genetically based mechanism of the regulation of amino acid metabolism [2].

Graph theory defines a number of network properties that allow successful analysis and interpretation of correlation networks (CN). These properties are a set of measures that describe the graph topology from different vantage points. CNs are undirected graphs, reflecting the coordinated behavior of two or more adjacent vertices (connected vertices) and the biological components they represent and not the effect of one vertex/component onto another, that is, a directed network. Properties that may have biological significance have been reviewed by Toubiana et al. [10]; they include (a)* vertex degree*: the number of edges incident on a given vertex [21], (b)* centrality score*: reflecting the number of shortest paths between a vertex and any other vertex in the network, (c)* network diameter*: the maximal shortest path between any two vertices in the graph, (d)* network density*: the ratio of existing edges to the number of all possible edges of a network, (e)* vertex betweenness centrality*: the relative number of the shortest paths between any two vertices that pass via a specific vertex, and (f)* modules*: subgraphs, within a global network characterized by higher connectivity (biologically interpreted as possible tighter coordination) between their components compared to other regions of the network. The analysis of these modules within the obtained network helped in the prediction of diseases [22, 23]. In this contribution we aim at providing an easy-to-implement pipeline for the generation of CNs for biologists without extensive computational skills. To do so, we are demonstrating the potential use of CNs in cancer studies.

Nowadays, there exist a number of software tools that allow researchers to generate networks, visualize them, and analyze their structure, via the calculation of a number of network properties, based on their own experimental data. Commonly known tools are Cytoscape [24], Gephi [25], and iGraph [26]. Each software has its benefits and disadvantages. For example, while iGraph requires programming skills and knowledge of the R programming language syntax, graphical-user-interface (GUI) based programs, such as Gephi and Cytoscape, do not, simplifying the interaction with the user. On the other hand, while script-based programs allow for the extension of existing functions and integration of compatible libraries, increasing the number of potential properties to be calculated, GUI programs are bound to the functionalities of the version of the software the researcher is using. However, Cytoscape and Gephi both offer a greater and easier-to-use set of visualization tools for networks, whereas the visualization functionalities of iGraph are rather limited and difficult to handle. Cytoscape allows for the integration of externally developed plugins, exerting functionality as desired by its developer. However, this option requires knowledge of the Java programming language and an understanding of how to interface it with the Cytoscape software.

The current proposed stepwise pipeline allows the user to control each step of the network creation, as it provides flexibility in selection of correlation methods and thresholds and describes easy-to-handle options to analyze the network topology. The pipeline works irrespective of the nature of the data set and can be implemented by a combined use of the freely distributed Apache OpenOffice software (http://www.openoffice.org/), built-in packages within the R-environment [27], and Cytoscape [24].

## 2. Method

**Figure 1:**
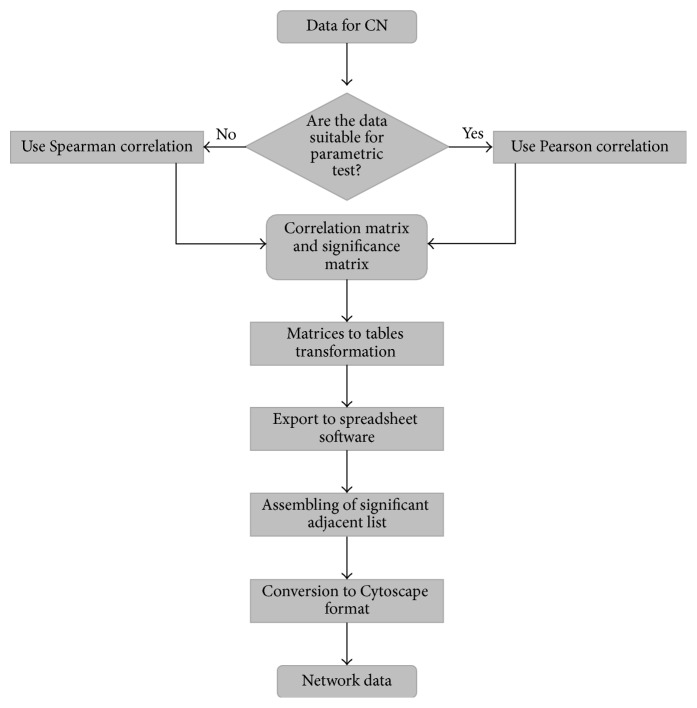
Correlation-based network, pipeline flowchart.

The construction of correlation-based networks starts form the calculation of the pairwise correlation coefficients between any two pairs of vectors of a given data set. One of the easiest ways to complete this calculation in big sets of data is to exploit the freely available R-software. There are several packages developed for correlation analysis under the R-environment. It is very important for the output matrix to select the proper type of correlation coefficient (Pearson, Spearman, Kendal, etc., represented as the letter “*r*”) and its corresponding thresholds (*r* and* p*). We recommend using the “*psych*” package under the R-environment [27, 28]. This package allows calculation of two diagonal matrices: (1) a symmetric diagonal* r-matrix* and (2) a symmetric diagonal* p-matrix*, where the lower triangle stores the *p*-*values* and the upper triangle the multiple hypotheses corrected *p*-*values*, corrected either by the Bonferroni correction or by applying a false discovery rate (FDR) correction. The obtained matrix with both* r*- and raw/adjusted *p*-*values* can be then transformed to the table view and exported to any spreadsheet software for a supervised selection of significant correlation coefficients. The thresholds of significance should be selected in respect to the nature and size of the data and considering the general suggestions as described in the introduction and elsewhere [29]. The selected significant correlation values can be easily converted to a table, listing in three columns the vertices that are adjacent to each other. This table is subsequently used as a template to illustrate the network using Cytoscape. We have chosen Cytoscape out of the list of network software as it was specifically developed for biological data, because of its intuitively understandable interface, wide range of visualization options, and available additional plugins for calculations of the main network properties. The method's workflow is presented in Figure 1.

### 2.1. Method Pipeline

#### 2.1.1. Download R-Environment and Required R-Packages

To start the workflow, first download and install the latest version of R-environment from the following website: https://www.r-project.org/. For the processes described here two R-packages will be used: “psych” [28] and “reshape2” [30]. Both packages are freely available for downloading via the R-environment window. As mentioned above, the R-environment is a freely available powerful statistical software often used to analyze biological data. Its benefits stem from the integration of various built-in functions and libraries/packages, supplemented by its ability to complement these by numerous externally developed packages and the freedom to combine them as necessary. Often, different packages offer different functions tackling the same task. For example, to compute correlation coefficients, one may use the core built-in function “cor” or the “rcorr” function of the Hmisc-package [31]. For the current work we have chosen specifically the “psych” package to perform correlation analysis as it conveniently computes the *r* coefficients and its corresponding *p* values and also performs* post hoc* tests to correct for multiple hypothesis testing (MHT). The package “reshape2” allows converting a matrix into a table and was chosen for this work for its easy implementation.

#### 2.1.2. Adjusting the Allocated Memory

Before beginning with the actual analysis, we recommend checking for the size of virtual memory available for R and Cytoscape, considering the potential large size of a data set. To do so for R under Windows OS type memory.limit() and if the result is smaller than the potential amount of your data set, increase the memory by typing memory.limit(size = 4096). This step allocates 4096 MB, equivalent to 4 GB (maximal number for 32 GB systems) of virtual memory, to the R-software. Unix-based OS's do not offer this function, as their virtual memory management is dynamic, adjusting itself to new and existing processes.

Similarly to the R-software the user may increase the memory allocated to Cytoscape, if, for instance, the size of a network is too large. Cytoscape is a Java-based software, so the first step here will be to access the* Configure Java* option via the Programs list. Next, select the* Java* tab in the displayed window, click on* View* button, and type* -Xms4096m* into the* Runtime parameters* line to allocated 4 GB of memory to the Cytoscape software. The amount of allocated memory is editable.

#### 2.1.3. Producing the Matrices (the R Code Necessary to Complete the Steps Described below Can Be Found in Supplementary Figure  1)

After the size of virtual memory is set, the user can start the pipeline according to the protocol presented in Supplementary Figure  1 available online at http://dx.doi.org/10.1155/2016/8313272. The described protocol represents a set of consequent commands (with an exception to the parallel computation of the* r-* and *p-value* matrices using the “psych” package), where the execution of one step is dependent on the former.

The output of the executed protocol will provide two separate files that can be opened in spreadsheet software. One of the files, “*r*_table.csv,” will represent a table view of the correlation matrix, and the second file, “*p*_table,” will represent the same table where* r-values* will be replaced by the correspondent *p values*. Probably the single disadvantage of this method is the time of calculation that strongly depends on number of the variables for the analysis and can be problematic for large (more than 500 variables) data sets. Nevertheless, the vast majority of metabolomics data sets does not exceed this amount of variables and usually is much smaller. Thus, the reader should not run into problems when executing the above code.

The obtained files “*r*_table.csv” and “*p*_table.csv” can be opened in any spreadsheet software (in our case OpenOffice). The next step is to remove the first column in each file and copy the rest to a new multisheet file on separate sheets for the* r-values* and the *p*-*values,* respectively. This step will provide two tables with two identical columns with the names of the variables, for example, metabolites/genes, and different third column with* r-* and *p*-*values,* respectively. At this stage the correlation threshold has to be selected.

#### 2.1.4. Selection of Significant Interactions and Arrangement of the Data to the Network Format Spreadsheet Software

Correlation coefficients,* r*, are the determining elements in CN construction; the threshold of acceptable *r*-value range and the threshold of its statistical significance will greatly affect the output of the network and its interpretation. The significance of a correlation is a two-factor concept. The first factor, the correlation coefficient (*r*), is expressed as a value ranging from −1 to 1, where positive and negative values represent a relation, alike or inverse, between the changes in the measure of the two variables. The magnitude of the coefficient reveals the strength of this relationship. However, the reliability of the model also depends on a second factor: the probability (*p*) of the detected* r-values*, reflecting a true relation. This value ranges from 0 to 1 and depends to a great extent on the sample size [32] but also on the experimental setup and the biological system of study. The selection of the threshold for both values depends largely on the researcher. It is trivial that *r* = 1 (perfect positive correlation) or *r* = −1 (perfect negative correlation) represent strong coordinated behaviors, while *r* = 0 shows the absence of a relation between the variables. But what can be said about intermediate* r's*? The “rule of thumb” suggests that there is no absolute* r*-threshold and different scientific disciplines apply different* r*-*value* thresholds. For example, in biology, thresholds from as low as |±0.3| have been proposed to be relevant, for example, for metabolic data in tomato introgression lines seeds and fruits [33], while in physics, an* r*-*value* lower than |±0.9| is often considered insignificant. Usually* r* ≥ |±0.5| is considered as “strong” by most of researches in biological systems [34]. The *p*-*value* that reflects significance of a correlation is usually accepted at three levels: 0.05, 0.01, and 0.001 [32]. However, since correlation analysis is applied on large data sets, *p*-*values* should usually be corrected by one of the* post hoc* tests for MHT, such as the Bonferroni correction or the false discovery rate (FDR) method, with the aim of avoiding false positives.

After both parameters of significance are decided, create a new sheet and copy the first two columns from any of the sheets (they are identical). In the first cell of the third column input the following formula:

(1) $$\begin{array}{c} =if\left(\;and\left(\;abs\left(\;X\;\right)>R,\;\;Y<P\;\right),1,0\;\right). \end{array}$$

In this formula *X* is the value of the 1st cell in the “*r*_values” sheet; *R* is the selected critical* r*-value; *Y* is the value of the 1st cell in the “*p*_values” sheet; *P* is the selected critical *p* value. Expand this formula to the whole table. This will provide an adjacency list that can be easily converted to the network format for Cytoscape software. For this, input in the next column following formula:

(2) $$\begin{array}{c} =if\left(\;X=1,concatenate\left(\;Y,\text{“}-\text{”},Z\;\right),\;\text{“}\;\text{”}\;\right). \end{array}$$

In this formula *X* is a number of 1st cell of the obtained column (usually 3rd) on the current sheet; *Y* and *Z* are the numbers of 1st cell in the 1st and 2nd column on the current sheet, respectively. At this stage copy 1st, 2nd, and 4th columns as the values to the new sheet, filter out and remove rows with empty last cell, and save the obtained fully filled three-column table in  .*txt tab delimited format*.

This file can be imported as a network to the Cytoscape software and analyzed using the built-in NetworkAnalyzer plugin. To import the file run the Cytoscape and select “*import*” from the main menu bar, then locate the previously saved three-column file in  .*txt* format, and import it as* “table”* (Supplementary Figure  2). To run the plugin locate* NetworlAnalyzer* in the Tool menu and execute* “Analyze Network*” (Supplementary Figure  3). The plugin will calculate the degree of vertices, vertex betweenness centrality, vertex clustering coefficient, and edge betweenness. The obtained parameters will be automatically added as attributes of vertices and edges of the network and can be visualized by customizable view options, including color, size, shape, and thickness. Additionally, NetworkAnalyzer can check if a vertex distribution of a network fits the power law, calculates the main properties of the network topology, such as diameter and global transitivity, and shows the average shortest path and other useful parameters.

## 3. Results and Discussion

Hypoxia is one of the major features of solid tumors affecting their development and treatment selection [35, 36]. The simulation of hypoxia in cancer cells* in vitro* can be used as a model study to understand the alteration of cancer cell metabolism that supports tumor growth under hypoxic conditions, the phenomena known as the “Warburg effect” [20, 37–39].

In short, the experiment included MDA-MB-231 breast adenocarcinoma cells that were incubated in 95% air and 5% CO_2_ at 37°C and 95% relative humidity and then were transferred to normoxic (21% oxygen) conditions. After 24 hours cell culture was divided into two groups, and one group was maintained under the normoxic condition, while the second group was transferred to a specific vessel with flow of gas containing 1% O_2_ and 5% CO_2_ balanced with N_2_ (hypoxic conditions). Next, GC-MS metabolic profiling of the two groups was performed [20].

**Figure 2:**
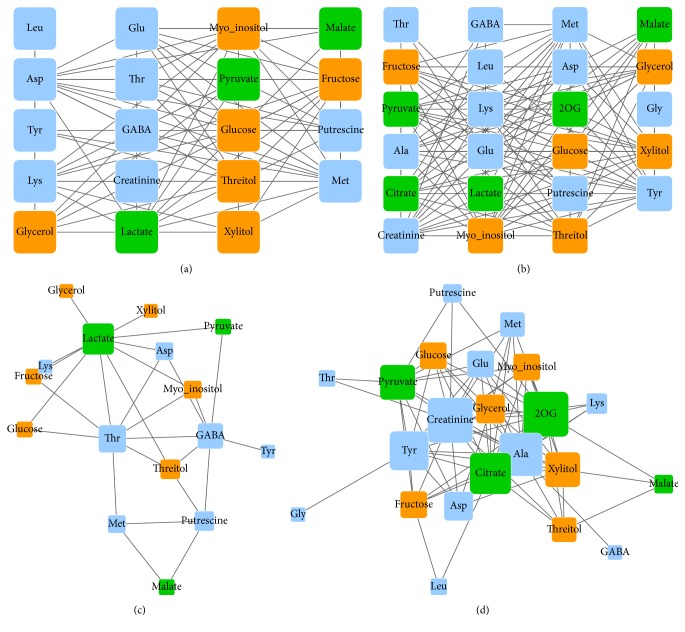
Correlation-based networks of metabolite data sets. (a) Original network under control conditions, (b) original network under hypoxic conditions, (c) network of unique relationships under control conditions compared to hypoxic conditions, and (d) network of unique relationships under hypoxic conditions compared to control conditions. Metabolic profiling of breast cancer cells under control and hypoxia (30 samples each) was used for pairwise correlation analysis between metabolites and network-view production. The data used to generate the network is from Kotze et al., 2013 [20]. Each vertex represents a metabolite; each edge represents a significant correlation between pairs of metabolites across samples. Vertex colors reflect biochemical classes: amino acids and N-compounds (blue), sugars and sugar alcohols (orange), and carboxylic acids (green). Vertex size reflects degree.

Prior to network construction, we first elaborated the published data, keeping uniquely identified metabolites only. Considering the sample size (*n* = 30) and the relatively large number of missing values in the data set, we decided to use Spearman's rank correlation with the thresholds *r* ≥ |±0.7| and *q*_FDR_ < 0.05. The applied procedure resulted in two adjacent tables (control (c) and hypoxia (h)) (Supplementary Data 1-2, control/hypoxia_adjacent_table resp. sheet) that were loaded to Cytoscape and visualized as a network (Figures 2(a) and 2(b), Supplementary Data 3). The simple comparison of two graphs revealed that the normal metabolic network was smaller compared to the network under hypoxic conditions. The differences in the number of vertices, *v*, were not very high (*v*_*c*_ = 19 versus *v*_*h*_ = 23, control versus hypoxia, resp.), but the number of edges, *e*, differed significantly (*e*_*c*_ = 87 versus *e*_*h*_ = 144, control versus hypoxia, resp.).

In order to identify metabolites or metabolic interactions specific to the hypoxic conditions, we used the “*merge*” tool in Cytoscape, selecting the two data networks. The tool gives multiple merging options visualizing either unique or common edges between two (or more) networks (Supplementary Data 3). The resulting merged graph displays common links (the union, for this kind of comparisons graph theory uses set-theory jargon). The comparison of the original graphs with the merged one is done by the same merging tool selecting the “difference” option and eventually it generates a graph (difference graph) for each comparison based on unique edges and vertices of the selected condition. The resulting difference graphs emphasize many condition-specific relations between metabolites existing in the two original networks (Figures 2(c) and 2(d)). The number of vertices changed to *v*_*c*_ = 16 and *v*_*h*_ = 22 for the control and the hypoxic conditions, respectively, and number of edges changed to *e*_*c*_ = 27 and *e*_*h*_ = 84, respectively. Thus, the gap (in folds) between the two *e* values increased from 1.67 to 3.1. The increased number of edges under hypoxia suggests the appearance of alternative metabolic routes to sustain the cell metabolism. Hypoxia treatment is used to mimic the conditions occurring in cancer cells because of high “uncontrolled” growth rate. Here, the unique metabolic relation identified could lead to the isolation of biochemical steps/reactions or common regulatory mechanisms between distantly related metabolites induced by the cancer growth (hypoxia treatment). Eventually the potential to identify markers defined as edges and not as vertices is significantly higher; just consider that the potential number of edges in a correlation (undirected) network with *n* metabolites can have *n∗*(*n* − 1)/2 interactions.

We then applied the NetworkAnalyzer plugin to calculate some of the topological properties of the networks such as network density, diameter, and transitivity and vertex degree and betweenness centrality (Tables 1, 2, and 3).

The results of the NetworkAnalyzer analysis of the networks topology suggested a reorganization of the metabolic network under hypoxia. Thus, a smaller (3 versus 4, Table 1) diameter, the longest shortest path between any two vertices in the network, and a larger (0.63 versus 0.38, Table 1) transitivity, the probability to form cliques in the network, suggest that the reorganization of the metabolic network under hypoxic conditions occurs via specific metabolites, namely, Ala, creatinine, 2OG, Tyr, and citrate. They act as hubs as they exhibit the greatest vertex degree and betweenness centrality measures (Table 3). In contrast, the properties of the network under normal conditions showed the topological importance of lactate, Thr, and GABA in Table 2. Surprisingly, lactate, the vertex with the highest betweenness centrality under normal conditions (0.46, Table 2), is absent in the hypoxia network (Figure 2(d)). This can be explained by the fact that nonoxidative metabolism is induced under stronger hypoxic conditions [20]. Alternatively, considering that lactate production is an indicator of inhibited respiratory [40], its absence in the correlation network under hypoxic conditions can suggest a strong specific effect of hypoxia on lactate irrespectively of other related metabolites. The results of GC-MS analysis revealed almost 1.3 times increase of lactate level under hypoxia compared to control conditions and support this suggestion [20]. Furthermore, the oxygen deficient condition leads not only to the increased conversion of glucose to lactate but also to the sharp suppression of citrate production [41]. The results of the GC-MS showed almost a twofold decrease in citrate levels under hypoxia compared to the control conditions [20]. The replacement of lactate to citrate in the metabolic network under hypoxic conditions, the high centrality of citrate in the network according to its vertex degree and betweenness centrality (Table 3), and the appearance of the citrate-2OG edge suggest the shift of citrate production from glucose oxidation to reductive carboxylation of 2OG (Figures 2(c) and 2(d) and Table 3) [41].

Glycolytic activity is high in cancer cells under both normal and hypoxic conditions. In the hypoxia network glycolysis derived pyruvate is strongly correlated with a row of biochemically related amino acids Ala, Asp, and Tyr, while in the network under normal conditions these associations were not detected (Figures 2(c) and 2(d)). Additionally, the unexpected drop of the correlation between pyruvate and GABA under hypoxia and the great centrality of Ala in the hypoxia network should be noted. GABA can be used in the transamination of pyruvate to produce alanine and succinic semialdehyde. GABA also accumulates under hypoxia in neurons of rats [42], and the present study shows that the level of GABA increased 1.5 times under hypoxia compared to control conditions [20]. Taken together these results suggest the transamination of pyruvate to Ala, possibly via GABA. Alternatively pyruvate is converted to Ala via alanine transaminase (ALT), involving Glu and 2OG (the latter also exhibiting a high centrality in the network), which act in a concerted action with aspartate transaminase (AST). The AST/ALT ratio in the blood of a human or animal is used in the diagnosis of liver damage or hepatotoxicity. By emphasizing the tight interaction between pyruvate, Ala, and Asp, our results likely show the metabolic reflection of a toxic condition imposed on the cell by hypoxia. Last, Ala is considered a marker of prostate [43] and breast [44] cancers where it significantly accumulates. However, the results by Kotze and coworkers did not reveal this in Ala level under hypoxia. We hypothesize that the changes in content of Ala might not be consistent between systems, while the actual coordinated response of Ala with a few tightly linked metabolites reflected within the network could potentially be a better candidate.

## 4. Conclusions

The interpretation of the CNs shows the relevance of graph theory in the analysis of biological data in general and specifically in the works dedicated to metabolic and genetic pathways. Implementing a network-based workflow using previously published data, we show how the pipeline can generate and visualize a network and how the network analysis can be used in biological studies. The presented pipeline aims at providing an easy to use but relatively powerful tool for* in silico* analysis of experimental data. The pipeline is not limited to metabolic data and can be effectively applied to gene coexpression network analysis, like the previously identified human disease-associated genes [45], lethal genes combination in yeast, and others [46–48]. This short essay exemplifies that the usage of CNs can lead to biologically sound conclusions on metabolic pathway regulation and original hypothesis generation without the need for complex and capacity consuming approaches. That said, CNs can be used as a part of top-down, complexity reduction approach leading to insights in the search and identification of marker genes or metabolites, respectively. Having said that, we wish to emphasize that the quality of the analysis more often than not depends on the design of the experiment and the sampling strategy.

## Supplementary Material

Supplementary Figure 1: Step-by-step protocol of correlation matrix calculation, significance test and transformation to an adjacent table under R-environment. Supplementary Figure 2: Data import in Cytoscape workspace. Supplementary Figure 3: Execution of NetworkAnalyzer in Cytoscape workspace. Supplementary Data 1: Network data output of metabolic analysis under control conditions. Data was previously published by Kotze et al. [20]. Supplementary Data 2: Network data output of metabolic analysis under hypoxic conditions. Data was previously published by Kotze et al. [20]. Supplementary Data 3: Complete correlation network analysis of metabolic data [20] in Cytoscape.

## Figures and Tables

**Table 1:** The main properties of the networks with unique interactions under control and hypoxia conditions, respectively.

Network name	Density	Diameter	Transitivity
Unique interactions under normal conditions (Figure 2(c))	0.23	4	0.38
Unique interactions under hypoxia conditions (Figure 2(d))	0.36	3	0.63

**Table 2:** The properties of the network of unique interactions under normal conditions in the descending order according to betweenness centrality. The compound class 1 represents amino acids, 2 sugars and sugar alcohols, and 3 carboxylic acids.

Metabolite	Compound class	Degree	Betweenness centrality
Lactate	3	10	0.460
Thr	1	8	0.255
GABA	1	7	0.224
Threitol	2	4	0.085
Putrescine	1	4	0.084
Met	1	3	0.065
Asp	1	3	0.015
Myo-inositol	2	3	0.015
Pyruvate	3	2	0.015
Fructose	2	2	0.000
Glucose	2	2	0.000
Glycerol	2	1	0.000
Lys	1	1	0.000
Malate	3	2	0.000
Tyr	1	1	0.000
Xylitol	2	1	0.000

**Table 3:** The properties of the network of unique interactions under hypoxia conditions in the descending order according to betweenness centrality. The compound class 1 represents amino acids, 2 sugars and sugar alcohols, and 3 carboxylic acids.

Metabolite	Compound class	Degree	Betweenness centrality
Ala	1	15	0.147
Creatinine	1	16	0.133
2OG	3	16	0.127
Tyr	1	13	0.113
Citrate	3	14	0.103
Pyruvate	3	11	0.060
Xylitol	2	11	0.037
Fructose	2	7	0.033
Threitol	2	6	0.010
Glycerol	2	8	0.006
Asp	1	8	0.005
Glucose	2	7	0.004
Met	1	6	0.004
Glu	1	7	0.003
Myo-inositol	2	7	0.003
Lys	1	4	0.001
Putrescine	1	3	0.001
GABA	1	1	0.000
Gly	1	1	0.000
Leu	1	2	0.000
Malate	3	3	0.000
Thr	1	2	0.000
